# A Support Vector Machine and Particle Swarm Optimization Based Model for Cemented Tailings Backfill Materials Strength Prediction

**DOI:** 10.3390/ma15062128

**Published:** 2022-03-14

**Authors:** Zhuoqun Yu, Yong Wang, Yongyan Wang

**Affiliations:** 1 College of Electromechanical Engineering, Qingdao University of Science and Technology, Songling Road No. 99, Qingdao 266061, China; yzqun2007@126.com; 2 School of Mechanical and Automation, Weifang University, Dongfeng East Road No. 5174, Weifang 261061, China; 3 School of Mechanical and Automotive Engineering, Qingdao University of Technology, Fushun Road No. 11, Qingdao 266033, China; wang_yong@qut.edu.cn

**Keywords:** cemented tailings backfill materials, unconfined compressive strength, machine learning, mechanical properties

## Abstract

This study aimed to investigate the feasibility of using a model based on particle swarm optimization (PSO) and support vector machine (SVM) to predict the unconfined compressive strength (UCS) of cemented paste backfill (CTB). The dataset was built based on the experimental UCS values. Results revealed that the categorized randomly segmentation was a suitable approach to establish the training set. The PSO performed well in the SVM hyperparameters tuning; the optimal hyperparameters for the SVM to predict the UCS of CTB in this study were *C* = 71.923, ε = 0.0625, and γ = 0.195. The established model showed a high accuracy and efficiency on the prediction work. The R^2^ value was 0.97 and the MSE value was 0.0044. It was concluded that the model was feasible to predict the UCS of CTB with high accuracy and efficiency. In the future, the accuracy and robustness of the prediction model will be further improved as the size of the dataset continues to grow.

## 1. Introduction

During the processing of mineral resources, a large amount of mine tailings was produced as a major byproduct. It is estimated that contemporary mine tailings production ranges between 5 and 7 billion tons per year worldwide [1]. However, most of the mine tailings were dumped into tailings dams, which caused a series of problems, such as land resources occupation and tailings dam failure [2]. Meanwhile, after mining an underground ore body, the mined-out stopes usually need to be backfilled [3]. Therefore, cemented tailings backfill (CTB) technology has been popular in recent years and has become an important way to dispose tailings [4,5,6].

Tailings, binder, and water were the main components of the CTB material [7]. After the mixing process, the CTB slurry was pumped into mine stopes through pipelines and cured for strength development during the hardening process [8]. During the service life of a CTB structure, it was important for the structure to have an acceptable mechanical strength to provide a stable support for the roof. One of the most important and useful mechanical properties for CTB design is the unconfined compressive strength (UCS). Several factors, such as the type of tailings, binder content, solid content, and curing age, could significantly affect the UCS of CTB [9,10]. Researchers and engineers have made great efforts to investigate the UCS of CTB in different situations; conducting a series of unconfined compression tests is the most common approach [11,12,13]. These experimental results have provided valuable information on the understanding of the strength development of CTB. However, experimental investigations were usually relatively time consuming. More efficient approaches need to be studied to help mining engineers rapidly estimate the UCS of CTB. Recently, several researchers have proposed various innovative approaches to predict the UCS of CTB. The basic theory was to find out the relationship between some values and UCS values, and thus to estimate the UCS of CTB based on the obtained values. For instance, Yilmaz and Ercikdi [14] used ultrasonic pulse velocity tests to predict the UCS of cemented paste backfill. They reported that there was a linear relation between the UCS values and ultrasonic pulse velocity. Xu et al. [15] used electrical resistivity measurement to assess the UCS growth of CTB material during the hydration process. They reported that the electrical resistivity properties were highly associated with the UCS and microstructural properties of cemented paste backfill material. Wang et al. [16] used viscosity to predict the UCS of CTB. They found that there was a positive linear relation between the viscosity and UCS of CTB. These researches have revealed the relationship between a certain experimental physical quantity and the UCS. However, it would further lead to greater efficiency gains and cost savings if the UCS of CTB could be predicted directly from the influence factors such as the type of tailings, binder content, solid content, and curing age. A major obstacle in capturing their relation is the significantly increasing complexity of the mathematical expressions when dealing with multidimensional and non-linear problems.

Fortunately, the development of artificial intelligence (AI) technology has provided efficient approaches to deal with the multidimensional and non-linear problems. In recent years, various AI algorithms have been used to build prediction models. For instance, some researchers have found that it was efficient to use support vector machine (SVM) to solve complex regression and classification problems [17,18,19]. In order to further improve the efficiency, some optimization algorithms were introduced and combined with the SVM. For instance, Olatomiwa et al. [20] hybridized the SVM with firefly algorithm (FFA) to predict the global solar radiation. The prediction model based on the hybrid machine learning algorithms proposed in their study was proved to be an efficient and accurate way to predict the global solar radiation. García Nieto et al. [21] hybridized the SVM with particle swarm optimization (PSO) to predict the remaining useful life of aircraft engines. They reported that the prediction model based on the hybrid PSO and SVM had good performance and dispensed with previous operation states information. However, few researchers have used the hybrid AI algorithms for UCS prediction. In 2018, Qi et al. [22] first used AI algorithms to predict the UCS of CTB materials. They combined PSO and Neural network to model the non-linear relationships between the influence variables and UCS of CTB materials. Three years later, they proposed an improved hybrid model based on adaptive neuro fuzzy inference system and artificial bee colony to predict the UCS, and they found the most significant influence variables for the UCS of CTB materials [23]. These results paved ways for the prediction of UCS of CTB materials using hybrid AI algorithms. Feasibility investigations on using more different AI algorithms, more performance improvement methods, and wider ranges of influence variables to predict the UCS of CTB are needed in order to further promote the practical application of AI technology for CTB design. Based on the previous studies on the AI prediction for UCS of CTB materials [22,23], curing time, cement-tailings ratio, and solid content were the most significant input parameters for the UCS of CTB. In addition, CTB with a lower binder content (3–10% of the mine tailings dry mass) gained popularity recently for its lower costs [24,25,26].

Therefore, this study aimed to investigate the feasibility of using a hybridized particle swarm optimization (PSO) and support vector machine (SVM) model to predict the UCS of CTB with lower binder contents and more curing ages.

## 2. Materials and Methods

### 2.1. Materials

**Figure 1:**
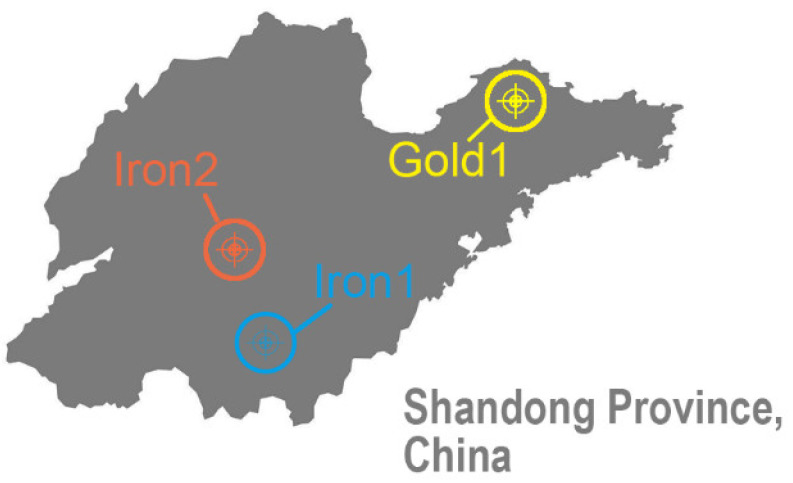
Sources of the tailings used in this study.

There were three types of tailings with different physical and mechanical properties used for the sample preparation, named as “Iron1”, “Iron2”, and “Gold1”. As shown in Figure 1, the three types of tailings used in this study were sampled from an iron mine in central Shandong Province, an iron mine in southern Shandong Province, and a gold mine in the northeastern Shandong Province, respectively.

In order to get the digital features for the machine learning approach, chemical and physical properties of the used tailings were measured using a laser particle size analyzer (Malvern, London, UK) and X ray fluorescence analyzer (Tianrui, Suzhou, China).

**Figure 2:**
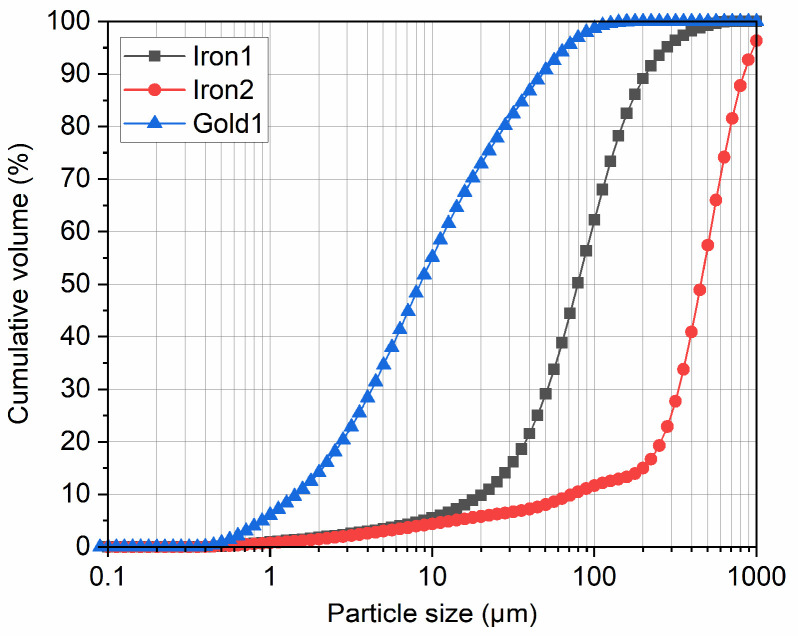
Particle size distribution of the tailings used in this study.

Figure 2 shows the particle size distribution of the three types of tailings. Table 1 shows the main physical and chemical properties of the three types of tailings obtained from the particle size distribution curves and XRF results.

P.O. 42.5 cement was used as the binder in this study. It was produced in Yangchun Cement Co. LTD, Zhucheng, China. Two cement contents (5% and 10% of dry tailings weight) were determined in this study according to the practical situation of cemented tailings backfill engineering [26,27,28,29]. City tap water that met the Chinese National Standards GB5749 [30] was used as the mixing water for the samples preparation. Main physical and chemical properties of the cement and water are shown in Table 2.

### 2.2. Preparation of Samples

Based on the common solid contents of CTB [31,32,33]. In this study, solid contents of these mixtures were determined to be 73%, 75%, 77%, and 79%. CTB samples were prepared using plastic molds and curing chamber. The mixing ratios of all CTB samples are shown in Table 3. First, dry tailings, cement and water were mixed in a laboratory mixer (Yitian, Hangzhou, China) for 7 min [34]. Then the mixtures were poured into the plastic cylindrical mold (50 mm in diameter and 100 mm in height) and left for 12 h. After being cured for 12 h, the samples were demolded and set in the curing chamber (Shouyi, Beijing, China) with a 95% relative humidity and 20 °C temperature. Curing ages were determined to be 3, 7, 14, and 28 days. At least two samples were prepared and tested for each mix ratio to ensure the repeatability of the results [35].

### 2.3. Unconfined Compressive Strength Tests

The unconfined compressive strength (UCS) test methods based on previous studies [26,36] and ASTM C39 [37] guidelines were carried out. A mechanical press system (Chaoyang tester manufacture, Chaoyang, China) was used as the test machine. Test samples that reached the specific curing age were placed axially between the bearing plates and loaded at a constant displacement rate of 0.2 mm/min. Peak strength values were recorded to obtain the UCS values. The experimental UCS values would be the core data of the dataset.

### 2.4. Machine Learning Algorithms

Two machine learning algorithms were used for predicting the UCS values of CTB. Support vector machine (SVM) was used as the predicting model and the radial basis kernel function (RBF) was determined to be the kernel function for SVM. SVM has lots of advantages when used for prediction and regression analysis, such as very little overfitting and effectiveness in high dimensional spaces [18,19,20,38,39]. However, the hyperparameters of SVM were very hard to tune manually, and the hyperparameters could determine the accuracy of the prediction model. Therefore, particle swarm optimization (PSO) was used to tune the hyperparameters for SVM. PSO is a nature-inspired optimization algorithm which was first built up by Kennedy and Eberhart [40]. It us a very effective optimization algorithm that has been used in many cases [22,41,42,43,44]. Introduction of the principles of PSO and SVM can be found in the previous study [45].

### 2.5. Establishment and Verification of the Prediction Model

**Figure 3:**
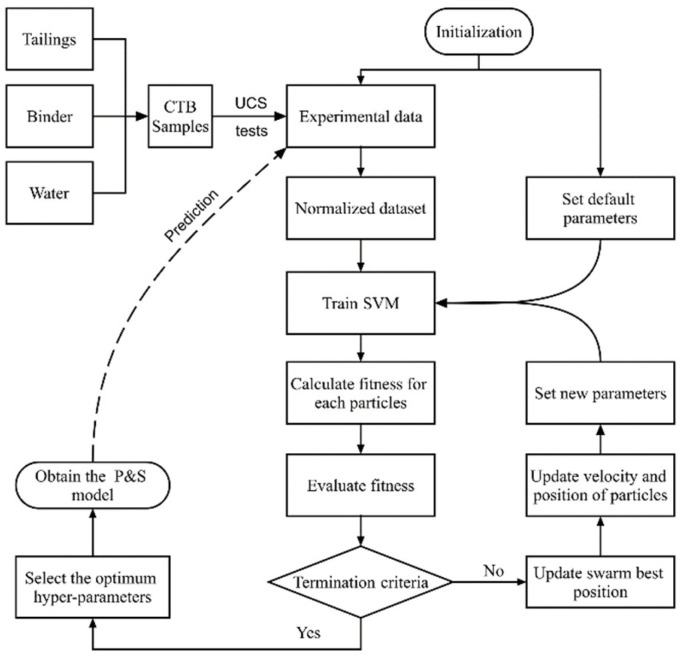
Flowchart of the P&S model for the UCS prediction.

The prediction model in this study was established by integrating the SVM and PSO algorithms. First, the SVM would be trained with default hyperparameters on the training set, then the PSO would tune the SVM hyperparameters in conjunction with the validation process. The 5-fold cross validation was used to validate its high accuracy and efficiency. The flowchart of the integrated PSO and SVM (P&S) model for the UCS prediction is shown in Figure 3. In this study, PSO parameters were determined based on trial tests and accumulated experience [21,22,38]. The swarm size and the maximum iteration were set to be 40 and 30 respectively. The *w*, *c*_1_, and *c*_2_ were set to be 0.5×ln2, 0.5+ln2, and 0.5+ln2, respectively. R-square (*R*^2^) and mean squared error (MSE) were used to verify the performance of the trained model.

## 3. Results and Discussion

### 3.1. Unconfined Compressive Strength Dataset

**Figure 4:**
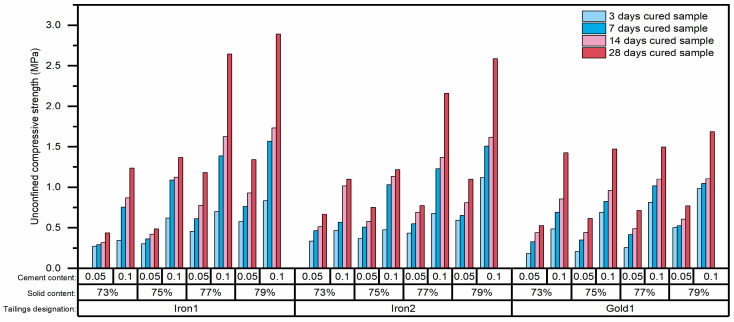
Unconfined compressive strength values of CTB samples.

The UCS values of CTB samples in this study are shown in Figure 4. It can be seen that the UCS increased with the increase of curing age. The CTB sample with a higher cement content had a higher UCS at the same curing age. These findings are likely to be related to the increased curing age and cement content leading to the formation of an increasing amount of cement hydration [12]. These results were consistent with the previous researches [29,46]. However, as there was no obvious law between the tailings type and the UCS value, it was hard to conclude or describe the effects of tailings type on the UCS of CTB by preliminary observation.

The dataset was built based on the experimental UCS values. The input variables were the curing age, cement content, solid content, and chemical-physical properties of tailings. The output variable was the UCS values. There were 1248 values (96 samples with 13 features) in this dataset. For instance, the 28 days cured CTB sample made of Iron2 tailings with 10% cement content and 75% solid content has 1 output value which is 1.22, and 13 input values which are 2.45, 1710, 73.17, 454.49, 850.14, 27.41, 8.894, 27.32, 12.93, 18.69, 0.1, 75, and 28. Units were ignored because all data would be scaled to the [–1, 1] range during the calculate process of P&S model.

### 3.2. Training and Verification of the P&S Model

#### 3.2.1. Overall Randomly Segmented Dataset

The whole dataset was segmented into two subsets, with a size ratio of 8:2 randomly to be the training set and test set, which is a common practice for predicting the UCS of different materials using artificial intelligence technology [22,45]. The training set was used for the P&S model training. The fitness values were recorded during the SVM hyperparameter tuning process by PSO. The ranges of the SVM hyperparameters are shown in Table 4.

**Figure 5:**
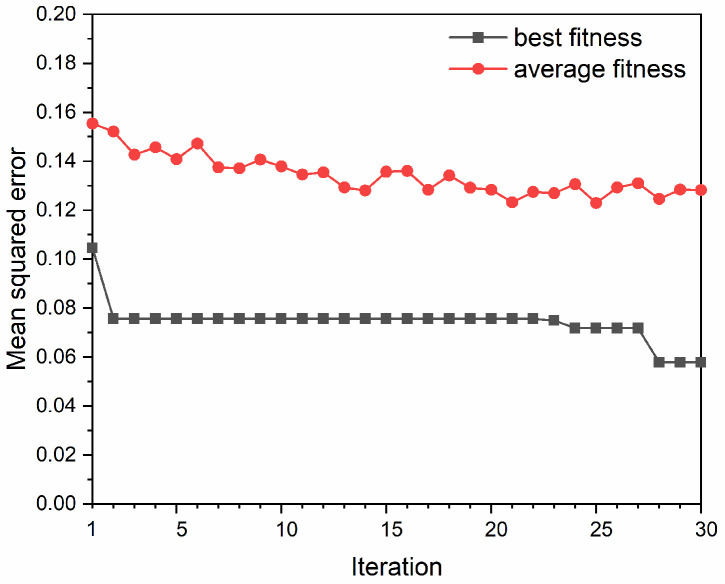
Best fitness and average fitness curves based on the overall randomly segmented dataset.

The fitness curves are shown in Figure 5. It can be seen that the best fitness decreased from 0.1046 to 0.0757 at the second iteration, and then kept constant until the 22nd iteration. After that, the best fitness decreased slightly and then kept nearly at 0.0578. It can also be seen that the average fitness showed a decrease trend before the 20th iteration, and then kept nearly at a constant. According to the tuning results, the optimal hyperparameters for the SVM prediction model were *C* = 78.349, ε = 0.0001, and γ = 0.595.

**Figure 6:**
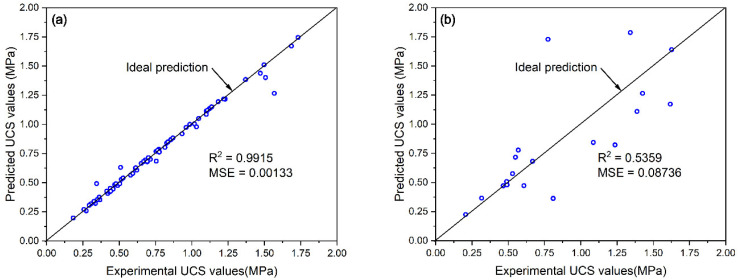
Prediction results based on overall randomly segmented dataset on (**a**) the training set and (**b**) the test set.

**Figure 7:**
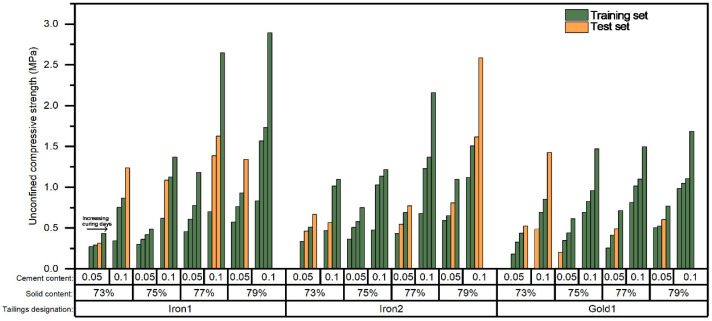
The specific data distribution of the training set and test set based on the overall randomly segmented dataset.

The prediction results and verification results on the training set and test set used the established P&S model based on the obtained hyperparameters are shown in Figure 6. It can be seen from Figure 6a that most predicted UCS values were almost equal to the experimental UCS values. The data points were concentrated near the ideal prediction line. Verification results also confirmed the high accuracy of this prediction model on the training set, as a high R^2^ value of 0.9915 and a good MSE value of 0.00133 were achieved. However, it can be seen from Figure 6b that the data points were scattered sparsely outside the ideal prediction line, which means that there were relatively large errors between the predicted values and the experimental values. A low R^2^ value of 0.5359 and a poor MSE value of 0.08736 were achieved, which indicated the poor accuracy of this prediction model on the test set. These results suggest that the obtained model may overfit the training set and lost the generalization. This may be due to the fact that the dataset contained four input variables from three different tailings; during the process of the training set establishment, the data were fetched randomly from the whole dataset and the random uniform dispersion of training data in different types of tailings was not considered enough. It can be seen from Figure 7 that the distribution of the training data in each type of tailings was not uniform. In addition, the dataset was relatively small due to the limitation of the experimental investigation. The common training set establishment approach based on the overall randomly segmented dataset may not achieve extensive uniform extraction when dealing with the prediction work for different types of tailings. This may cause the overfitting and result in the poor accuracy.

#### 3.2.2. Categorized Randomly Segmented Dataset

**Figure 8:**
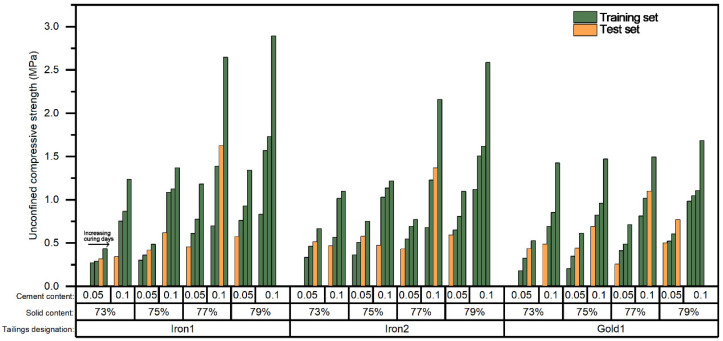
The specific data distribution of the training set and test set based on the categorized randomly segmented dataset.

The P&S model was aimed to predict the UCS value of different tailings. Since the dataset was composed of three different types of tailings experimental data, the whole dataset was first segmented into three subsets according to the classification of the tailings. Then each of the subsets was segmented into two subsets with a size ratio of 8:2 randomly to be the training set and test set. Figure 8 shows the details of the training set and test set based on the categorized randomly segmented dataset. It can be seen that the data of the training set and the test set achieved a uniform distribution in each type of tailings.

**Figure 9:**
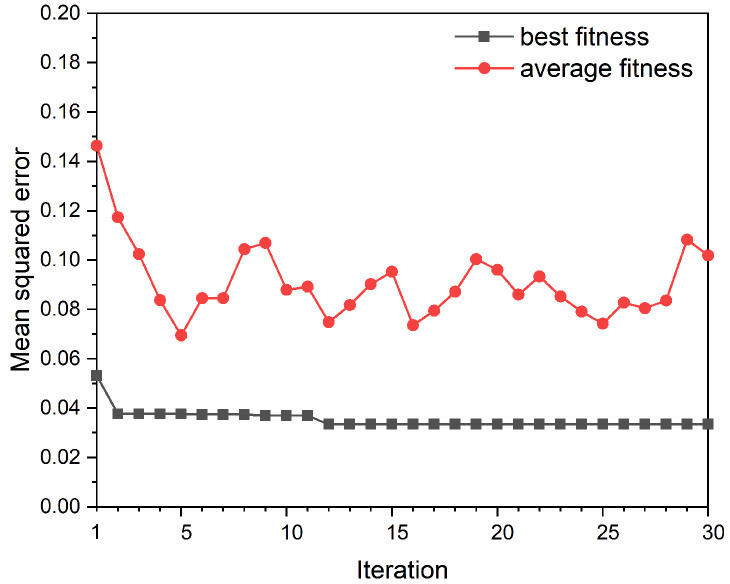
Fitness curves based on the categorized randomly segmented dataset.

The hyperparameters tuning process of the P&S model based on the categorized randomly segmented dataset can be exhibited by the fitness curves as shown in Figure 9. The ranges of the SVM hyperparameters are shown in Table 3. It can be seen that the best fitness decreased from 0.0532 to 0.0335 before the 12th iteration. The average fitness decreased continuously before the fifth iteration and then fluctuated around 0.09. The tuning results showed that the optimal hyperparameters for the SVM were *C* = 71.923, ε = 0.0625, and γ = 0.195.

**Figure 10:**
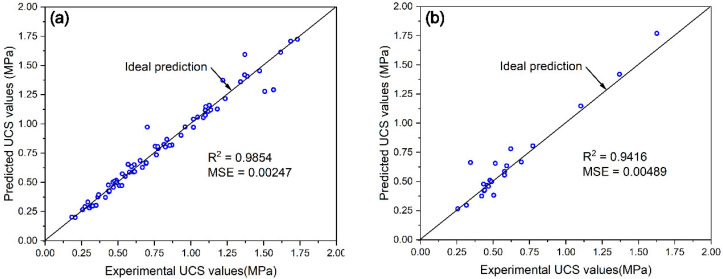
Prediction results based on categorized randomly segmented dataset on (**a**) the training set and (**b**) the test set.

Based on the obtained hyperparameters, the P&S model was trained on the training set and tested on the test set. The prediction results and verification results are shown in Figure 9. It can be seen from Figure 10a that the model performed well in the training set. A high R^2^ value of 0.9854 and a good MSE value of 0.00247 were achieved and most prediction data were almost equal to the experimental data. It can also be seen from Figure 10b that the data points were concentrated near the ideal prediction line, and a high R^2^ value of 0.9416 and a good MSE value of 0.00489 were achieved. This indicated that the model had been trained well and achieved high accuracy on both the training set and the test set. The categorized randomly segmentation may be a suitable way to train the model for predicting the UCS of different types of tailings backfill materials.

### 3.3. Prediction Capability of the P&S Model

**Figure 11:**
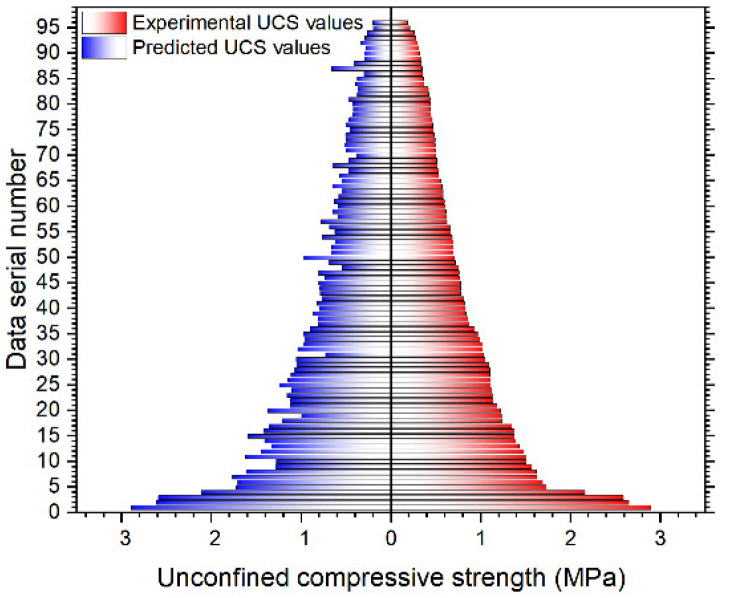
Comparison of UCS values obtained from the experimental investigation and P&S model prediction.

After the well trained P&S model was obtained, the whole experimental data were used to test the capability of the P&S model on the UCS prediction. Figure 11 intuitively shows the comparison between the experimental UCS values obtained from the unconfined compression test and the UCS values predicted by the trained P&S model. On the right half of Figure 11, the experimental UCS data are arranged in descending order from the largest to the smallest. The predicted UCS data on the left half of the figure correspond to the experimental data on the right half of the figure. It can be seen that most predicted UCS values were similar to the experimental UCS values. The shape consisting of the predicted and experimental UCS value bars is basically symmetrical. The R^2^ value was 0.97 and the MSE value was 0.0044, which indicated a high prediction accuracy of the P&S model. The whole computation time for the prediction work was 4 s, using MATLAB 2017a on a personal computer with Intel Core i5 processor. This indicates the high efficiency of the P&S model.

It can also be seen from Figure 11 that several predicted UCS values have relatively large errors. This may have been due to the inadequate training caused by the relatively small size of the dataset. Future research should be undertaken to obtain more experimental UCS values with more refined curing ages and mix material proportioning settings. Denser data points will allow for better training of the P&S model, which could lead to a higher accuracy achievement.

In this study, UCS of CTB samples made of three types of tailings were tested and used for the training and verification of machine learning prediction work. However, in order to achieve the UCS prediction for CTB with any types of tailings, more CTB samples made of different types of tailings should be prepared and tested to let the prediction model find out the relationship between the physical and chemical properties of tailings and the UCS of CTB, to thus improve the robustness. An online database that can be accessed by researchers around the world could be very helpful to rapidly expand the size of the dataset [23].

As a result, the P&S model had a good capability of predicting the UCS of CTB with different types of tailings, different curing ages, and different mixing proportioning settings. In the future, the accuracy and robustness of the prediction model will be further improved as the size of the dataset continues to grow. This could be helpful to assist the CTB design process with a high efficiency and accuracy.

## 4. Conclusions

The current study investigated the feasibility of using an AI prediction model based on P&S to predict the UCS of CTB with lower binder contents. Based on the results, we offer the following conclusions:

(1) The common approach for the establishment of training set based on the overall randomly segmented dataset may cause overfitting and result in the poor accuracy (a low R^2^ value of 0.5359 and a poor MSE value of 0.08736 were achieved on the test set) when dealing with the prediction work for CTB with different types of tailings.
(2) The categorized randomly segmentation may be a suitable way to train the model for predicting the UCS of different types of tailings backfill materials. Compared to the overall randomly segmentation approach, a much higher R^2^ value of 0.9416 and a better MSE value of 0.00489 were achieved on the test set after the training of the P&S model.
(3) PSO performed well in the SVM hyperparameters tuning. The optimal hyperparameters for the SVM to predict the UCS of CTB in this study were *C* = 71.923, *ε* = 0.0625, and *γ* = 0.195.
(4) The P&S model showed high accuracy and efficiency on the prediction work. The R^2^ value was 0.97, the MSE value was 0.0044, and the whole computation time was 4 s. It is feasible to use the P&S model to predict the UCS of CTB with lower binder contents, different curing ages, and different types of tailings.

This study provided an approach based on the P&S model to predict the UCS of CTB. However, due to the limitation of obtaining a wider variety of tailings, only three types of tailings were used to train the model. In the future, more types of tailings will be used to train the model and new types of tailings could be used for the verification of the trained model.

## Figures and Tables

**Table 1:** Main physical and chemical properties of the tailings.

Physical Properties	Iron1	Iron2	Gold1	Chemical Composition	Iron1	Iron2	Gold1
Specific gravity	2.76	2.45	2.81	SiO_2_	55.50	27.41	64.64
Specific surface area (cm^2^/g)	2640	1710	1600	Al_2_O_3_	2.93	8.89	16.79
D10 (μm)	20.41	73.17	1.58	Fe_2_O_3_	23.80	27.32	4.84
D50 (μm)	79.62	454.49	8.93	MgO	3.18	12.93	1.14
D90 (μm)	208.89	850.14	50.23	CaO	5.26	18.68	2.72

**Table 2:** Main physical and chemical properties of the cement and water.

Physical Properties	Cement	Water	Chemical Composition	Cement (%)	Water (mg/L)
Specific gravity	3.10	1.00	SiO_2_	21.40	-
D10 (μm)	6.66	-	Al_2_O_3_	4.31	-
D50 (μm)	33.2	-	Fe_2_O_3_	4.91	-
D90 (μm)	81.2	-	MgO	3.00	-
			CaO	62.34	-
			CaCO_3_	-	80.00
			Sulphate	-	32.80
			Chloride	-	21.34
			Fluoride	-	0.40
			Nitrate	-	2.18

**Table 3:** Mixing ratios of all CTB samples.

Tailings Type	Cement Content ^a^ (%)	Solid Content ^b^ (%)	Curing Ages
Iron1, Iron2, Gold1	5	73	3, 7, 14, 28
Iron1, Iron2, Gold1	10	73	3, 7, 14, 28
Iron1, Iron2, Gold1	5	75	3, 7, 14, 28
Iron1, Iron2, Gold1	10	75	3, 7, 14, 28
Iron1, Iron2, Gold1	5	77	3, 7, 14, 28
Iron1, Iron2, Gold1	10	77	3, 7, 14, 28
Iron1, Iron2, Gold1	5	79	3, 7, 14, 28
Iron1, Iron2, Gold1	10	79	3, 7, 14, 28

^a^ Relatively to the dry tailings weight; ^b^ Relatively to the dry tailings + cement weight.

**Table 4:** SVM hyperparameters and their tuning ranges.

Hyperparameters	Explanation	Range
*C*	Penalization parameter	1–100
*ε*	Insensitivity	0.0001–0.1
*γ*	Parameter of the kernel function	0.001–100

## Data Availability

The datasets used or analyzed during the current study are available from the corresponding author upon reasonable request.
